# Combined cerium and zinc oxide nanoparticles induced hepato-renal damage in rats through oxidative stress mediated inflammation

**DOI:** 10.1038/s41598-023-35453-5

**Published:** 2023-05-25

**Authors:** Olola Esther Adeniyi, Olayinka Anthony Adebayo, Oluyemi Akinloye, Oluwatosin Adekunle Adaramoye

**Affiliations:** 1 Present Address: Department of Biochemistry, Faculty of Basic Medical Sciences, College of Medicine, University of Ibadan, Ibadan, Nigeria; 2 Department of Biochemistry, University of Ibadan, Ibadan, Nigeria; 3 Clinical Chemistry and Molecular Diagnostic Laboratory, Department of Medical Laboratory Science, Faculty of Basic Medical Sciences, University of Lagos, Lagos, Nigeria; 4 Biochemistry Programme, College of Agriculture, Engineering and Science, Bowen University, Iwo, Osun State Nigeria

**Keywords:** Biochemistry, Biotechnology

## Abstract

The toxicity profiles of nanoparticles (NPs) used in appliances nowadays remains unknown. In this study, we investigated the toxicological consequences of exposure to cerium oxide (CeO_2_) and zinc oxide (ZnO) nanoparticles given singly or in combination on the integrity of liver and kidney of male Wistar rats. Twenty (20) rats were allotted into four groups and treated as: Control (normal saline), CeO_2_NPs (50 μg/kg), ZnONPs (80 μg/kg) and [CeO_2_NPs (50 μg/kg) + ZnONPs (80 μg/kg)]. The nanoparticles were given to the animals through the intraperitoneal route, three times per week for four repeated weeks. Results revealed that CeO_2_ and ZnO NPs (singly) increased serum AST and ALT by 29% & 57%; 41% & 18%, and co-administration by 53% and 23%, respectively. CeO_2_ and ZnO NPs increased hepatic and renal malondialdehyde (MDA) by 33% and 30%; 38% and 67%, respectively, while co-administration increased hepatic and renal MDA by 43% and 40%, respectively. The combined NPs increased hepatic NO by 28%. Also, CeO_2_ and ZnO NPs, and combined increased BAX, interleukin-1β and TNF-α by 45, 38, 52%; 47, 23, 82% and 41, 83, 70%, respectively. Histology revealed hepatic necrosis and renal haemorrhagic parenchymal in NPs-treated rats. Summarily, CeO_2_ and ZnO NPs produced oxidative injury and induced inflammatory process in the liver and kidney of experimental animals.

## Introduction

Among the various divisions in nanotechnology, nanomedicine stands out as the fastest emerging field. This is due to the ability of engineered nanoparticles to be deplored as alternative therapy in the management of pathological conditions, with minimal adverse reactions^1^. Engineered nanoparticles have improved characteristic features such as shape, distribution, binding affinity and size, which is different from that of the parent compounds^2^. In recent years, there has been outstanding advancement in nanotechnology in which nanoparticles has been synthesised and engineered to perform specific functions^3^. Nanoparticles have found application in medicine and other day-to-day activities such as in optical imaging of major cellular components, important component of surgical sutures, delivery of drugs to target organs and fabric improvement^4,5^.

Cerium and zinc oxide nanoparticles are two important particles that have received enormous biomedical attention in recent years due to their biocompatibility and antioxidative properties^6,7^. Zinc, an essential element in the biological system serves as co-factor for a number of enzymes such as peptidases and anhydrases^8^. In humans, an approximately 2 to 3 g zinc uptake is required out of about 12 mg daily need of zinc^9^. The small size of zinc renders it easily absorbed from zinc oxide nanoparticles, which takes part in regulating physiological processes in the body^10^. The biomedical application of zinc oxide on nanoscale formulations has been studied and research is still on-going^11^. The low toxicity of zinc oxide nanoparticles to the skin has enabled it to be used as an important component of sunscreen creams, where it has been proposed to act as protection against ultraviolent radiations from the sun^12^. Also, zinc oxide nanoparticles (ZnONPs) has been shown to possess antibacterial actions against a number of bacteria, such as *E. coli* and *Salmonella* species of various morphological identities^13^. In addition, recent reports have demonstrated that zinc oxide nanoparticles may play essential role in drugs and gene delivery^14^. Cerium oxide nanoparticles (CeO_2_NPs) or nanoceria may be considered as one of the essential nanoparticles having applications in fuel cells, oxygen pumps, gas sensors, and as a fertilizer in Chinese agriculture^15^. The self-generating ability of cerium oxide nanoparticles by reversibly inter-switching between oxidation states of + 3 and + 4 renders it an important therapeutic agent. In contrast to other nanoparticles, the cerium oxide in nano state maintains its fluorite configuration, thereby forming an oxygen vacancy in its lattice^16^. The vacant oxygen so form renders it more catalytically active and thus its antioxidant potentials^17^. The biomedical application of cerium oxide nanoparticles includes its ability to reduce tumor volume and weight in experimental rats as well as its cytotoxic, pro-apoptotic, and anti-invasive capacity on melanoma cells^18^. The mechanism of antitumor activity of cerium oxide nanoparticles has been traced to its antioxidant activity against reactive oxygen species (ROS)^19^. Recent studies have found strong association between exposure to ultra fine nanoparticles and adverse health effects in humans^20,21^. Due to their unique physical and chemical characteristics, nanoparticles have been the focus of much research owning its industrial applications and environmental toxicity^22^. The biomedical and industrial applications of these nanoparticles has therefore increased the possibility of human exposure to more than one nanoparticles per time and their resultant effect might posse more harm than good to public health. Therefore, this study was designed to evaluate the toxic effects of cerium and zinc oxide NPs, given singly or combined on the hepato-renal system of male Wistar rats.

## Materials and methods

### Chemicals

Glutathione (GSH), hydrogen peroxide (H_2_O_2_), adrenaline and trichloroacetic acid (TCA) were purchased from Sigma-Aldrich (St. Louis, MO, USA), while 2-thiobarbituric acid (TBA), *O*-dianisidine and dithionitrobenzoic acid (DTNB) were obtained from British Drug House (BDH), Poole, UK. Other reagents and chemicals used were of analytical quality.

### Nanoparticles

The nanoparticles (CeO_2_NPs) and (ZnO NPs) used during this study were obtained from Greg Goss Research Group, University of Alberta, Edmonton, Canada. The cerium oxide and zinc oxide nanoparticles are negatively charged with an average diameter less than 10 nm and 13 nm respectively. They were functionalized with poly acrylic acid polymer coating to render it highly dispersible even in suspension. The unique features of the particles in solution have been highly characterized and described by^23^.

### Ethical approval

Before the commencement of this study, approval was obtained from the Animal Care and Use Regulatory Committee (ACUREC), University of Ibadan, Nigeria and the body endorsed the experimental procedures with approval number UI-ACUREC/18/0039. The study was carried out in accordance with relevant guidelines and regulations in handling of experimental animals, where they were treated with the least discomfort. The study is reported in accordance with ARRIVE guidelines.

### Treatment of animals

Mature male Wistar rats (140.00 ± 5.20 g) were acquired from Animal breeding division of the Faculty of Basic Medical Sciences, University of Ibadan, Nigeria. The animals were housed in properly aerated cages and maintained at ambient temperature (25 °C) under a regulated 12:12 light/dark phases. They were nourished with standard laboratory feed and water ad libitum.

### Experimental design

The animals were allowed to acclimatize for two weeks and thereafter, twenty (20) rats were assigned into four groups of five animals each. The first group (control) received normal saline, while other groups received cerium oxide nanoparticles (CeO_2_NPs) at 50 μg/kg body weight, zinc oxide nanoparticles (ZnONPs) at 80 μg/kg body weight and [CeO_2_NPs (50 μg/kg) + ZnONPs (80 μg/kg) ] via intraperitoneal route. The route and dosage were decided according to the studies of^24,25^, respectively. The nanoparticles were given thrice per week for four consecutive weeks. The reason for administering thrice per week and not daily was to prevent bioaccumulation of the nanoparticles which may contribute to toxic responses that will be beyond the scope of the research. The choice of 50 μg/kg CeO_2_ NPs was due to the previous result from our laboratory where we used graded doses (100 μg/kg, 200 μg/kg and 300 μg/kg) of CeO_2_ NPs and result showed that these doses induced organ toxicity Adebayo et al. (2018). We therefore decided to check if exposure to lower doses would be safe. The reason for choosing 80 μg/kg ZnO NPs dose?

### Preparation of tissue and serum

Twenty-four hours after the administration of last dose of CeO_2_NPs and ZnONPs, food was withdrawn from the animals overnight, anesthetized (using ketamine at 35 mg/kg) and sacrificed by cervical dislocation the next day. The liver and kidney were carefully excised, rinsed in ice cold buffer solution (1.15% KCl) and blotted. The weight of the organs was determined and recorded. Part of the whole liver was cut, and one of the pair of the kidneys were kept in 10% buffered formalin. The remaining portions of liver and whole kidney were homogenized in phosphate buffer (50 mM & pH 6.8). The resulting mixture was centrifuged at 10,000*g* for 15 min to obtain post-mitochondrial fraction (PMF). Blood was collected from rats via retro-orbital bleeding into plain bottles, allowed to clot and centrifuged at 3000*g* for 15 min to obtain serum. Both serum and post mitochondria fraction, PMF were used for biochemical analyses.

### Biochemical assays

#### Determination of alanine and aspartate aminotransferases (ALT & AST), and total bilirubin

Activities of ALT, AST and level of total bilirubin in the serum were evaluated using the method described by^26–28^, respectively.

##### Aspartate aminotransferase activity

Diluted sample (100 µL) was combined with Aspartate (0.1 mL), sodium phosphate buffer solution (0.1 mL of 100 mmol/L at 7.4 pH), and 2 mL of α-oxoglutarate to make the reacting mixture. This was followed by thirty minutes of incubation at a temperature of 37 °C. Later on, a volume of 500 µL of 2, 4-dinitrophenylhydrzine was introduced into the reacting mixture and left on the bench for about 20 min at a temperature of 25 °C. An additional (5.0 mL) NaOH was introduced, and then the optical density (546 nm) was recorded alongside the (reagent) blank within 5 min.

##### Alanine aminotransferase activity

Diluted sample (100 µL) was reacted with l-alanine (0.1 mL), sodium phosphate buffer solution (0.1 mL of 100 mmol/L at 7.4 pH), and 2 mL of α-oxoglutarate to make the reaction mixture. This was followed by 30 min of incubation at a temperature of 37 °C. Later on, a volume of 500 µL of 2, 4-dinitrophenylhydrzine was introduced into the reaction mixture and left on the bench for about 20 min at a temperature of 25 °C. An additional (5.0 mL) NaOH was introduced, and then the optical density (546 nm) was recorded alongside the (reagent) blank within 5 min.

##### Determination of total bilirubin

Briefly, the reaction mixture contained 200 μL of sample, 200 μL of a solution of sulfanilic acid (29 mmol/L) and hydrochloric acid (0.17 N), 50 μL of sodium nitrite (38.5 mmol/L), and 1.0 mL of caffeine (0.26 nmol/L) and sodium benzoate (0.52 mol/L), which were incubated at room temperature for 10 min. After incubation, 1.0 mL of tartrate (0.93 mol/L) and sodium hydroxide (1.9 N) were added and the mixture was incubated for 10 min at room temperature. The absorbance was read against a blank at 546 nm.

#### Determination of urea and creatinine levels

Serum urea and creatinine levels were assessed by the methods of^29,30^ respectively.

#### Protein determination

Serum and PMF protein levels were determined according to the method of^31^ using bovine serum albumin (BSA) as standard. In brief, 0.5 mL diluted sample/ standard was added to 0.7 mL Lowry reagent [Mixture of alkaline solution (NaOH and Na_2_CO_3_)] and was incubated for 20 min at room temperature. Thereafter, 0.1 mL of diluted Folin’s reagent (2N Folin and Ciocalteu’s phenol reagent, and 6 mL distilled water) was added, vortex mixed and incubated for 30 min. After incubation, absorbance was taken at 750 nm and protein values were estimated by extrapolation from BSA calibration curve.

#### Determination of catalase (CAT) activity

Catalase activity was determined by the method of^32^. The mixture of 2.4 mL of phosphate buffer (50 mM, pH 7.0), 1.0 mL of 19 mM H_2_O_2_ and 50 µL of sample were incubated at room temperature for 3 min. Next, the reaction was terminated by the addition of 2 mL of dichromate/acetic acid solution, followed by heating in boiling water bath for 10 min. The solution was cooled at room temperature and, the decrease in absorbance was measured in a spectrophotometer at 570 nm for 3 min. Catalase activity was expressed as Unit/mg protein.

#### Assessment of the activity of superoxide dismutase (SOD)

Activity of SOD was determined according to the method of^33^. The method is based on the inhibition of autoxidation of epinephrine (pH 10.2) at 30 °C. The assay mixture contained 50 µL of the sample, and 2.5 mL of 0.05 M carbonate buffer (pH 10.2). Freshly prepared 0.3 mL of 0.3 mM adrenaline was added and mixed by inversion. The increase in absorbance at 480 nm was monitored spectrophotometrically at 30 s intervals for 150 s. The specific activity of SOD was expressed in Units/mg protein.

#### Estimation of the activity of glutathione-*s*-transferase (GST)

The activity of GST was determined by the method of^34^ using CDNB as a substrate. The reaction mixture is made up of 1.7 mL of 100 mmol/L phosphate buffer (pH 6.5) and 0.1 mL of 30 mmol/L of CDNB. After pre-incubating the reaction mixture at 37 °C for 5 min, the reaction was started by the addition of 20 µL of sample, and absorbance was followed for 5 min at 340 nm. Reaction mixture without the enzyme served as a blank. Specific activity of GST was expressed as micromole of GSH/CDNB conjugate formed per min per mg protein using an extinction coefficient of 9.61 per mmol per cm.

#### Assessment of the activity of glutathione peroxidase (GPx)

The GPx activity was determined by the method of^35^ The reaction mixture contained 500 µL of sodium phosphate buffer, 100 µL of 10.0 mM of sodium azide, 200 µL of 4.0 mM of reduced glutathione (GSH), 100 µL of 2.5 mM of H_2_O_2_ and 50 µL of the sample. This was made up to 2.0 mL with distilled water and incubated for 3 min at 37 °C. The reaction was terminated by the addition of 0.5 mL of 10% trichloroacetic acid and centrifuged. The supernatant obtained was used for the determination of residual GSH by the addition of 4.0 mL of disodium hydrogen phosphate (0.3 mM) solution, and 1.0 mL of 5,5’’-dithio-bis-2-nitrobenzoic acid (DTNB) reagent. The absorbance was measured in a spectrophotometer at 412 nm and GPx activity was expressed as micromole/mg protein.

#### Estimation of reduced glutathione (GSH) level

Level of GSH was determined according to the method of^36^. Briefly, an aliquot of PMF was deproteinized by the addition of an equal volume of 4% sulfosalicylic acid, and the resulting solution was centrifuged at 3000*g* for 15 min at 4 °C. Supernatant (100 µL) was then added to 1.5 mL of DTNB. The GSH was proportional to absorbance at 412 nm. Values were expressed in micromole/g tissue.

#### Assessment of lipid peroxidation level

The amount of lipid peroxide formed in the liver and kidney was estimated by the method of^37^. Briefly, 0.4 mL of sample was reacted against 1.6 mL of Tris-KCl solution containing 0.5 mL 30% TCA. Subsequently, 0.5 mL of 0.75% TBA solution was added to the mixture and placed into a boiling water bath at 90 °C for a period of 45 min. The mixture was left to cool and then centrifuged for 15 min at 3000*g*. The optical density of the supernatant was measured using a spectrophotometer at 532 nm against a blank.

#### Determination of markers of apoptosis and inflammation

##### (a) Interleukin-1β (IL-1β), tumour necrosis factor alpha (TNF-α), BAX, caspases-3 and-9

Serum levels of IL-1β and TNF-α (Markers of inflammation) and BAX, caspases-3 and-9 (Markers of apoptosis) were determined using commercial ELISA kits (Ray-Biotech incorporation) by following the manufacturer’s instructions.

##### (b) Estimation of nitric oxide (NO) level

The concentrations of NO_3_^−^ and NO_2_^−^ in the serum, liver and kidney were estimated as an index of NO production by the method of^38^. The amount of nitrite in the serum, renal and hepatic PMF was estimated following Griess’ reaction. In the reaction mixture, 0.5 mL of sample was incubated with 0.5 mL of Griess’ reagent at 37 °C for 20 min. The absorbance was measured in a spectrophotometer at wavelength 550 nm, and the concentration of nitrite was assessed by comparing the resulting optical density with a standard curve of well-known sodium nitrite concentration.

##### (c) Estimation of the activity of myeloperoxidase (MPO)

The activity of myeloperoxidase (MPO) was assessed by the modified method of^39^. The hepatic and renal MPO activities were estimated using spectrophotometry technique by reacting *O*-dianisidine with hydrogen peroxide. Myeloperoxidase, a lysosomal enzyme catalyzes the oxidation of *O*-dianisidine in the presence of H_2_O_2,_ acting as an oxidizing agent to yield a brown-coloured product, which absorbs maximally at wavelength 470 nm.

### Statistical analysis

The results were presented as mean ± standard deviation. Data were analyzed by one-way analysis of variance (ANOVA) using SPSS version 23.0 (SPSS Inc., Chicago, IL, USA). For the post Hoc test, the Least Significant Difference (LSD) was used and the differences between control and test groups were considered significant at *p-*values less than 0.05.

## Results

### Effect of cerium and zinc oxide NPs on liver biomarkers in Wistar rats

Table 1 showed that rats administered CeO_2_ and ZnO NPs singly or combined had insignificant (p > 0.05) increase in the organo-somatic weight of liver relative to the control group. The activities of serum AST and ALT significantly (p < 0.05) increased in animals administered CeO_2_ and ZnO NPs singly as well as the combined group (Table 2). Precisely, AST activities increased by 29%, 59% and 53% in rats administered CeO_2_ and ZnO NPs, and combined group, respectively. Similarly, ALT activities increased by 41% and 23% in rats administered CeO_2_NPs and [CeO_2_NPs + ZnONPs], respectively.

**Table 1:** Effect of cerium and zinc oxide nanoparticles on body and organs’ weight of Wistar rats.

Treatments	Body weight (g)			Organs’ weight (g)		Relative weight (%)	
	Initial	Final	Weight gain (g)	Liver	Kidney	Liver	Kidney
Control	139.4 ± 1.95	207.6 ± 15.0	68.2 ± 8.0	7.73 ± 0.0	1.43 ± 0.1	3.72 ± 0.2	0.69 ± 0.0
CeO_2_NPs	145.6 ± 1.52	223.4 ± 15.7	77.8 ± 5.0(14 ) ↑	9.90 ± 0.3*(28 ) ↑	1.45 ± 0.2(1 ) ↑	4.43 ± 0.1(19 ) ↑	0.65 ± 0.2(6 **)**↓
ZnONPs	160.2 ± 3.27	239.8 ± 6.9	79.6 ± 9.2(17 ) ↑	9.96 ± 0.1*(29 ) ↑	1.53 ± 0.1(7 ) ↑	4.15 ± 0.2(12 ) ↑	0.64 ± 0.1(5 ) ↓
CeO_2_NPs + ZnONPs	161.0 ± 2.00	225.8 ± 13.7	64.8 ± 6.3(4 ) ↓	9.70 ± 0.0*(25 ) ↑	1.65 ± 0.2(15 ) ↑	4.30 ± 0.1(16 ) ↑	0.73 ± 0.2(6 ) ↑

Values are expressed as mean ± S.D of 5 animals.

*CeO*_*2*_*NPs* cerium oxide nanoparticles,* ZnONPs* zinc oxide nanoparticles.

*Significantly different from control (p < 0.05). ↓ signifies decrease while ↑ signifies increases.

**Table 2:** Effect of cerium and zinc oxide nanoparticles on serum hepatic and renal biomarkers in Wistar rats.

Treatments	Liver			Kidney	
	AST (U/L)	ALT (U/L)	Bilirubin (U/L)	Creatinine (μmol/L)	Urea (μmol/L)
Control	101.6 ± 6.8	111.7 ± 2.4	17.8 ± 1.5	389.9 ± 10.1	242.7 ± 1.4
CeO_2_NPs	143.6 ± 5.6*(29)↑	187.5 ± 3.5*(41) ↑	19.2 ± 0.4(6) ↑	395.5 ± 1.4(1 ) ↑	223.7 ± 6.5(9 ) ↓
ZnONPs	250.8 ± 12.6*(59 ) ↑	122.5 ± 3.5(9 ) ↑	13.5 ± 9.2(37)↓	391.4 ± 1.4(1 ) ↑	203.9 ± 1.4(19 ) ↓
CeO_2_NPs + ZnONPs	217.3 ± 14.6*(53 ) ↑	145.6 ± 4.8*(23 ) ↑	16.5 ± 1.2(11 ) ↓	398.4 ± 5.8(2 ) ↑	190.2 ± 0.7(27 ) ↓

Values were expressed as mean ± S.D of 5 animals.

*CeO*_*2*_*NPs* cerium oxide nanoparticles, *ZnONPs* zinc oxide nanoparticles.

*Significantly different from control (p < 0.05), ↓ signifies decrease while ↑ signifies increase.

### Effect of cerium and zinc oxide NPs on inflammation & oxidative stress markers in rats

**Figure 1:**
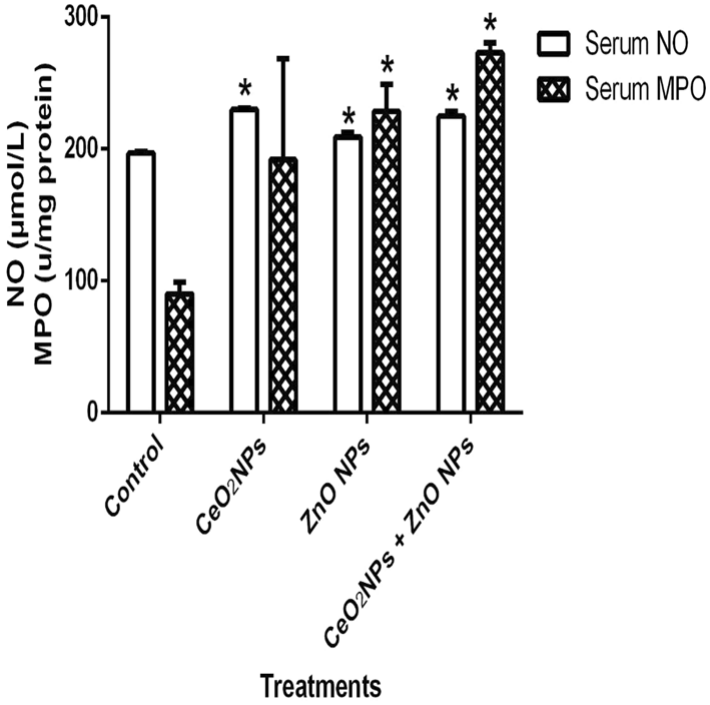
Effect of cerium and zinc oxide nanoparticles on the levels of serum nitric oxide and activities of myeloperoxidase in male Wistar rats showing significant increase in serum nitric oxide and myeloperoxidase across the treatment groups compared to control. *Significantly different from control (p < 0.05). *CeO2NPs* cerium oxide nanoparticles, *ZnO NPs* zinc oxide nanoparticles.

**Figure 2:**
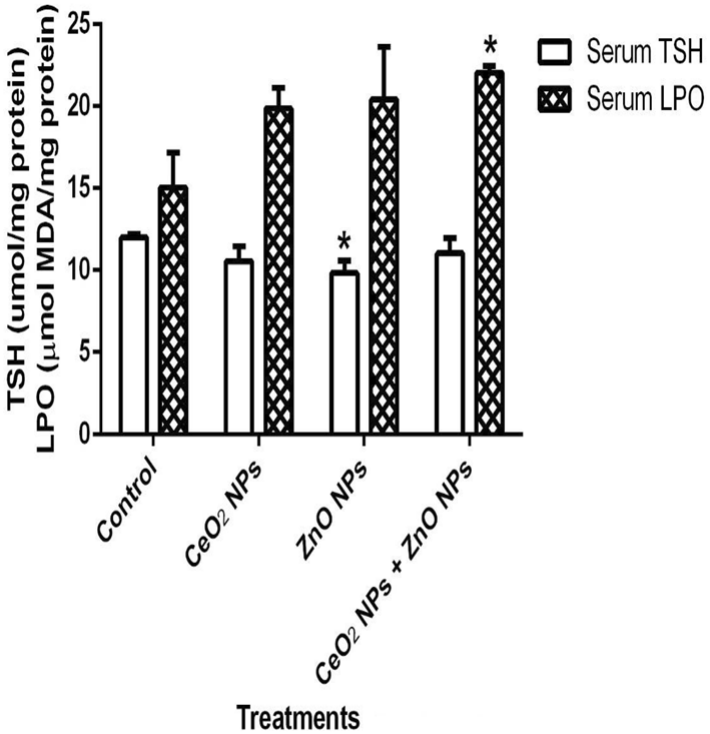
Effect of cerium and zinc oxide nanoparticles on the levels of serum total sulphydryl and lipid peroxidation in male Wistar rats showing significant decrease in serum TSH in the group treated with cerium oxide nanoparticles and significant increase in serum LPO of groups administered the combined nanoparticles. *Significantly different from control (p < 0.05). *CeO2NPs* cerium oxide nanoparticles, *ZnONPs* zinc oxide nanoparticles.

**Figure 3:**
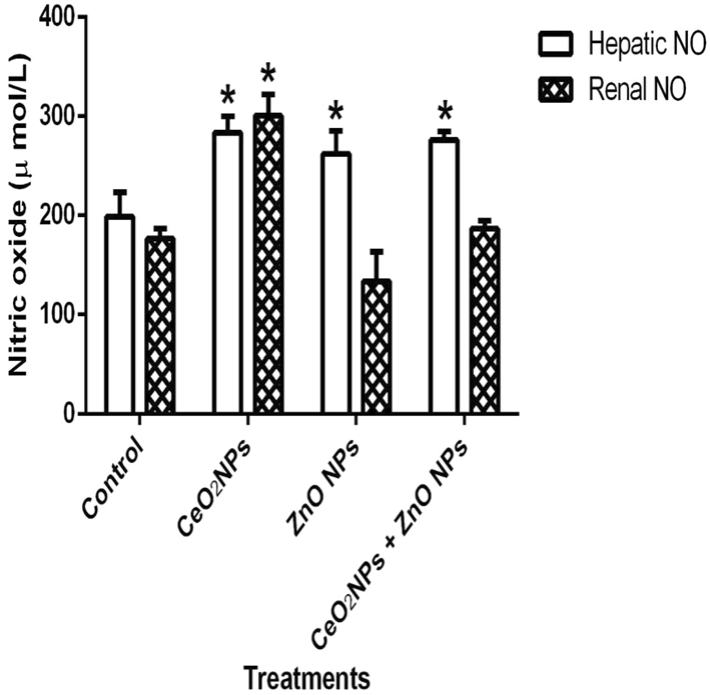
Effect of cerium and zinc oxide nanoparticles on hepatic and renal nitric oxide level in male Wistar rats showing significant increase in hepatic nitric oxide across the treated groups while renal nitric oxide increased significantly in the cerium oxide nanoparticles group. *Significantly different from control (p < 0.05) *CeO2NPs* cerium oxide nanoparticles, *ZnONPs* zinc oxide nanoparticles.

**Figure 4:**
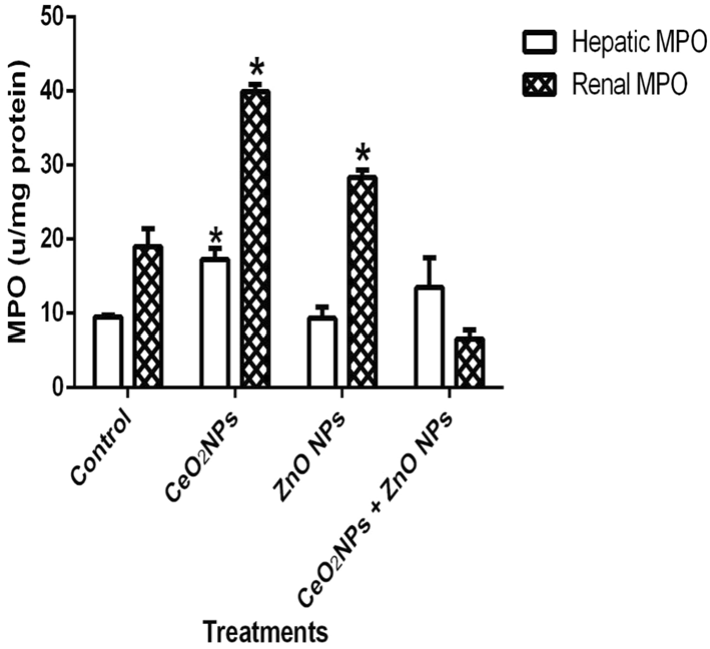
Effect of cerium and zinc oxide nanoparticles on hepatic and renal myeloperoxidase activities in male Wistar rats showing significant increase in renal myeloperoxidase activities in cerium oxide and zinc oxide nanoparticles administered singly while hepatic myeloperoxidase increased significantly only in the cerium oxide nanoparticles administered groups. *Significantly different from control (p < 0.05). *CeO2NPs* cerium oxide nanoparticles, *ZnONPs* zinc oxide nanoparticles.

**Figure 5:**
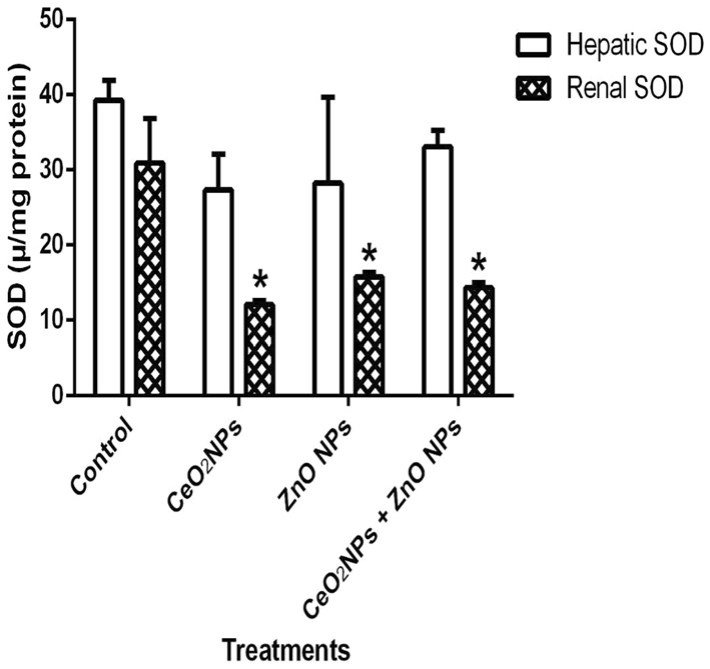
Effect of cerium and zinc oxide nanoparticles on hepatic and renal activities of superoxide dismutase in male Wistar rats showing significant decrease in renal superoxide dismutase activities across the treatment groups when compared to the control group, while there was no difference in hepatic superoxide dismutase across all treatment groups. *Significantly different from control (p < 0.05). *CeO2NPs* cerium oxide nanoparticles, *ZnONPs* zinc oxide nanoparticles.

**Figure 6:**
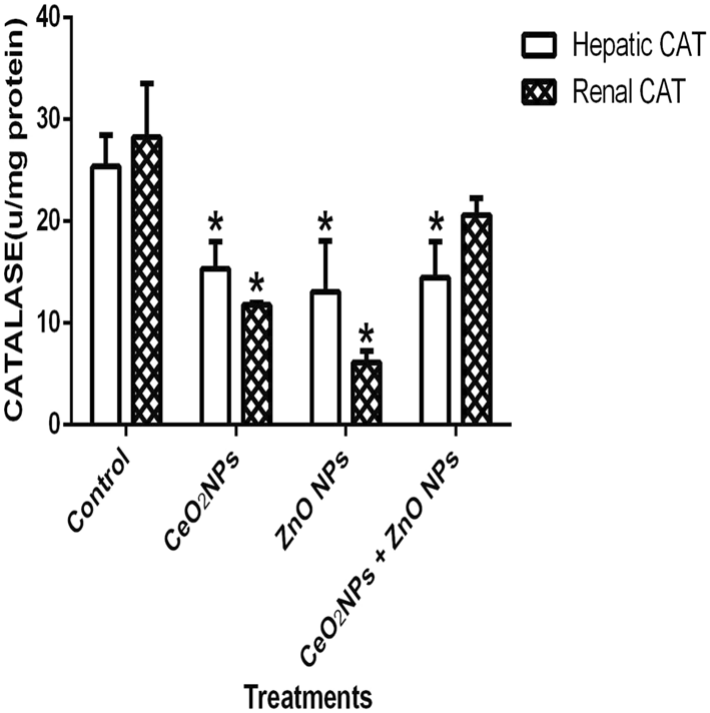
Effect of cerium and zinc oxide nanoparticles on hepatic and renal activities of catalase in male Wistar rats showing significant increase in hepatic and renal catalase activities across all the treated groups when compared to the control group. *Significantly different from control (p < 0.05). *CeO2NPs* cerium oxide nanoparticles, *ZnONPs* zinc oxide nanoparticles.

**Figure7:**
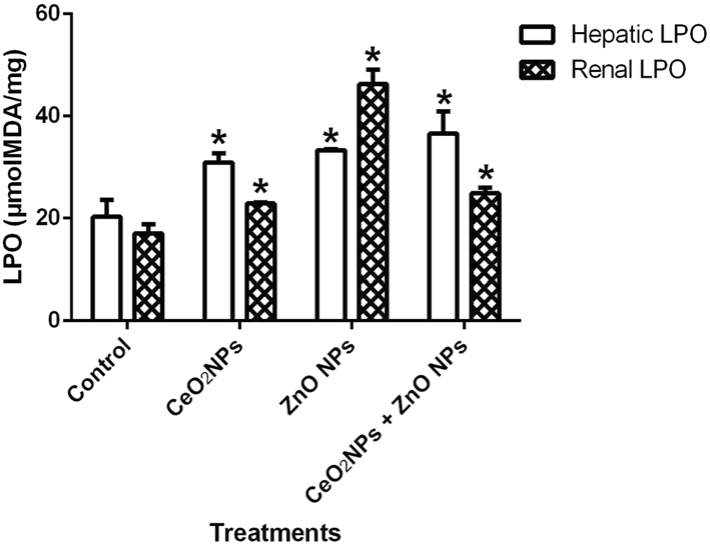
Effect of cerium and zinc oxide nanoparticles on hepatic and renal levels of lipid peroxidation in male Wistar rats, showing a significant increase in peroxidation of the liver and kidney of groups of animals administered with cerium oxide nanoparticles, zinc oxide nanoparticles singly and in combination. *Significantly different from control (p < 0.05). *CeO2NPs* cerium oxide nanoparticles, *ZnONPs* zinc oxide nanoparticles.

**Figure 8:**
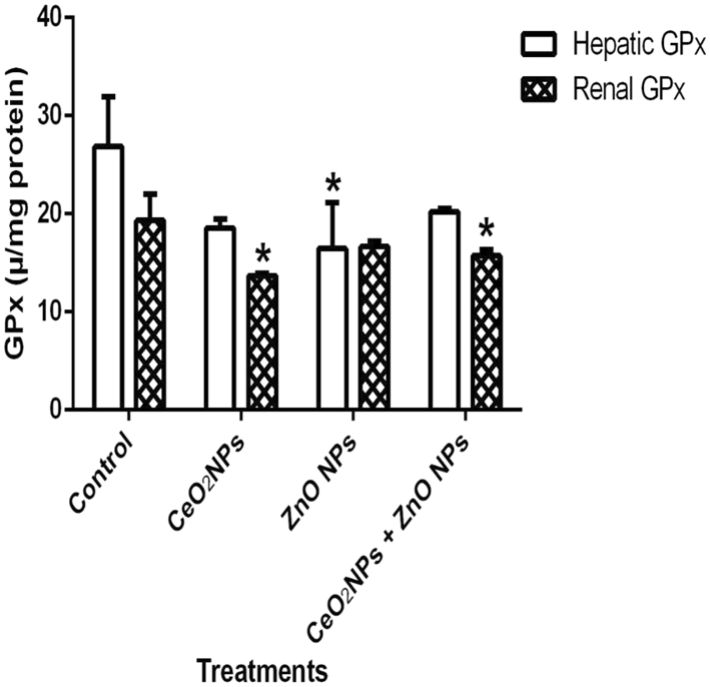
Effect of cerium and zinc oxide nanoparticles on hepatic and renal activities of glutathione peroxidase in male Wistar rats showing significant decrease in renal glutathione peroxidase activities in groups administered cerium oxide nanoparticles singly and combined with zinc oxide nanoparticles, while hepatic glutathione peroxidase decreased significantly only in the zinc oxide nanoparticles group. *Significantly different from control (p < 0.05). *CeO2NPs* cerium oxide nanoparticles, *ZnONPs* zinc oxide nanoparticles.

**Figure 9:**
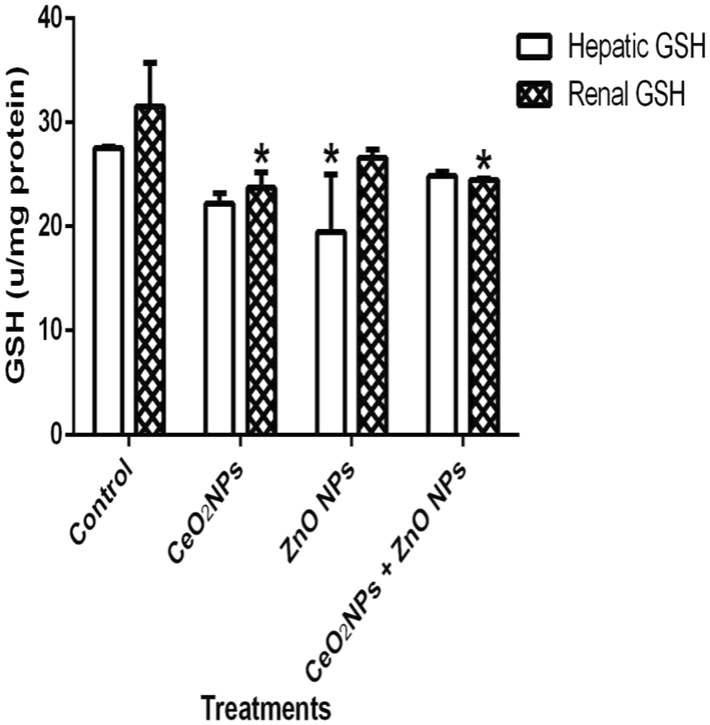
Effect of cerium and zinc oxide nanoparticles on hepatic and renal levels of reduced glutathione in male Wistar rats showing significant decrease in hepatic reduced glutathione in groups administered with cerium oxide nanoparticles singly and combined with zinc oxide, while renal reduced glutathione decreased only in the zinc oxide nanoparticles group. *Significantly different from control (p < 0.05). *CeO2NPs* cerium oxide nanoparticles, *ZnONPs* zinc oxide nanoparticles.

In Fig. 1, levels of serum nitric oxide (NO) and activities of myeloperoxidase increased significantly (p < 0.05) in rats administered CeO_2_ and ZnO NPs singly, and in combined group. Likewise, the levels of serum lipid peroxidation products increased by 34%, 36% and 48% in animals given CeO_2_ and ZnO NPs singly, and combined group, respectively. In addition, levels of lipid peroxidation in the serum increased by 33%, 34% and 47% in animals given CeO_2_ and ZnO NPs singly, and combined group respectively. (Fig. 2). However, insignificant (p > 0.05) decrease in the levels of serum total sulphydryl was observed in the NPs administered groups when compared to the control, with the exception of ZnO NPs where there was 33% decrease in levels of total sulphydryl. Furthermore, hepatic NO increased by 40, 36 and 38% in rats administered CeO_2_ and ZnO NPs singly, and combined group, respectively, while renal NO increased by 37% in animals given CeO_2_NPs alone (Fig. 3). In Fig. 4, activities of renal MPO increased by 98 and 46% in rats administered CeO_2_ and ZnO NPs singly, respectively, while hepatic MPO increased by 76% in animals given CeO_2_NPs alone. In Figs. 5 and 6, administration of CeO_2_ and ZnO NPs singly, and combined for four consecutive weeks caused significant (p < 0.05) decrease in the activities of renal and hepatic SOD and catalase of the animals. The decrease in the activities of these first line antioxidant enzymes was accompanied by significant (p < 0.05) increase in the levels of renal lipid peroxidation (LPO) products by 40, 118 and 46% and hepatic LPO by 48, 52 and 58%, respectively in animals given CeO_2_ and ZnO NPs singly, and combined (Fig. 7). In addition, Figs. 8 and 9 showed that antioxidant parameters; hepatic and renal activities of GPx and the levels of GSH were significantly (p < 0.05) decreased in animals administered these NPs either singly or in combination. In addition, inflammatory markers; IL-1β and TNF-α increased by 47, 23, 82% and 41, 83, 70% in rats treated with CeO_2_ and ZnO NPs singly, and combined, respectively (Table 3).

**Table 3:** Effect of cerium and zinc oxide nanoparticles on the levels of serum BAX, interleukin-1β (IL-1β) and TNF-α in male Wistar rats.

Treatment	IL-1β (pg/mL)	TNF-α (pg/mL)	BAX (ng/mL)
Control	84.0 ± 14.4	223.2 ± 17.5	12.8 ± 2.4
CeO_2_NPs	123.6 ± 20.1* (46 ) ↑	315.1 ± 28.4* (41) ↑	18.6 ± 1.3* (45) ↑
ZnONPs	103.2 ± 18.4* (22 ) ↑	407.6 ± 23.9* (82 ) ↑	17.7 ± 2.9* (38) ↑
CeO_2_NPs + ZnONPs	153.1 ± 22.0* (82 ) ↑	380.1 ± 18.1* (73 ) ↑	19.5 ± 2.2* (52) ↑

Values are expressed as mean ± S.D of 5 animals.

*CeO*_*2*_*NPs* cerium oxide nanoparticles, *ZnONPs* zinc oxide nanoparticles.

*Significantly different from control (p < 0.05). ↑ signifies increase and ↓ signifies decrease.

### Effect of cerium and zinc oxide NPs on apoptosis & cyto-architecture of tissues of rats

**Figure 10:**
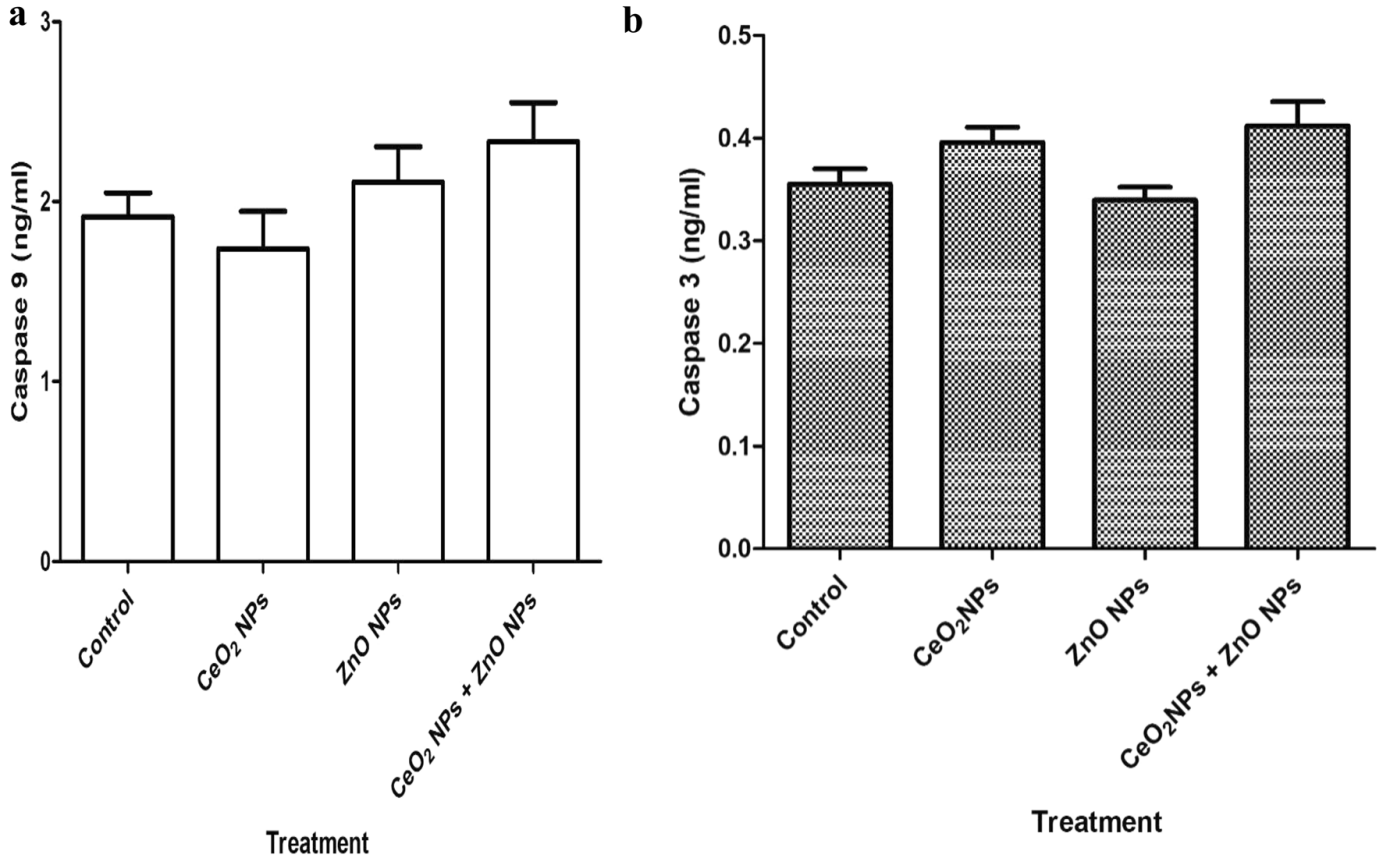
(**a**) Effect of cerium and zinc oxide nanoparticles on serum caspase 9 level in male Wistar rats revealed as light decrease in caspase 9 in groups treated with cerium oxide nanoparticles and as light increase in zinc oxide nanoparticles and the combined nanoparticles. *CeO2NPs* cerium oxide nanoparticles, *ZnONPs* zinc oxide nanoparticles. (**b**) Effect of cerium and zinc oxide nanoparticles on serum level of caspase 3 in male Wistar rats, showing slight increase in cerium oxide nanoparticles singly and combined nanoparticles, while there was no difference in the group treated with zinc oxide nanoparticles. *CeO2NPs* cerium oxide nanoparticles, *ZnONPs* zinc oxide nanoparticles.

**Figure 11:**
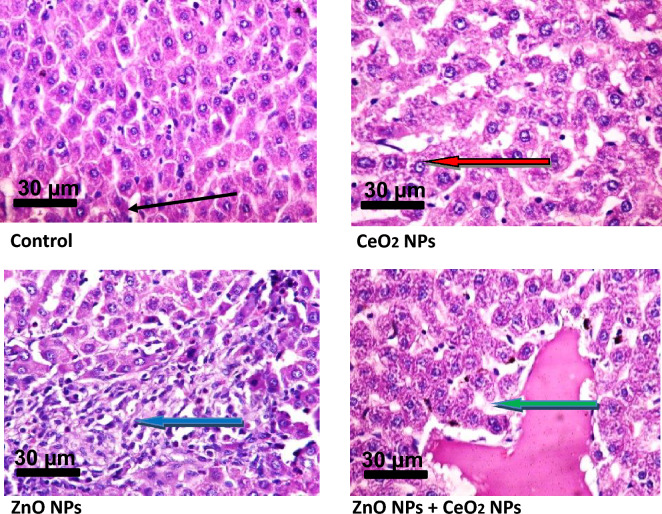
Photomicrograph showing the effect of cerium and zinc oxide nanoparticles on the integrity of hepatocytes in male Wistar rats. In the control, the sinusoids appear normal (slender arrow) without infiltration of inflammatory cells, CeO2 NPs and ZnO NPs groups shows necrosis (reds arrow)] and infiltration of inflammatory cells (blue arrow) respectively, while in the ZnO NPs + CeO2 NPs group, the liver parenchyma show severely dilated sinusoid with congestion (green arrow).

**Figure 12:**
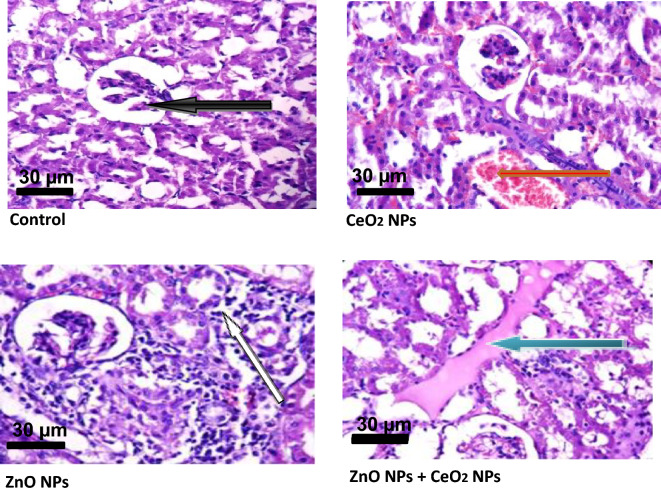
Photomicrograph showing the effect of cerium and zinc oxide nanoparticles on the integrity of renal tissues in male Wistar rats. In the control, the renal cortex shows normal glomeruli with normal messengial cells and capsular spaces (black arrow), while in CeO2 NPs group, the interstitial spaces shows mild haemorrhage (red arrow). In the ZnO NPs group, the renal cortex show severe periglomerular infiltration of inflammatory cells (red arrow) while ZnO NPs + CeO2 NPs group, the interstitial spaces show moderate congestion (green arrow).

In Table 3, administration of CeO_2_ and ZnO NPs singly and combined, increased the levels of serum BAX (Marker of apoptosis) by 45, 38 and 52%, respectively. Figure 10a, b showed insignificant (p < 0.05) increase in the levels of caspases-3 and-9 in rats administered CeO_2_ and ZnO NPs singly and combined. Figures 11 and 12 show representative photomicrographs of liver and kidney of rats administered CeO_2_ and ZnO NPs singly and combined. In the controls, the tissues displayed normal architectural arrangement with little or no observable lesions. However, in rats treated with CeO_2_ and ZnO NPs singly, and combined, hepatic necrosis and infiltration of inflammatory cells were observed. These observations were more pronounced in animals treated with ZnONPs and combined group. Also, rats given CeO_2_ and ZnO NPs singly, and combined showed tubular degeneration in renal medulla and congestion of interstitial blood vessel.

## Discussion

This study confirmed via biochemical and histological means that CeO_2_ and ZnO nanoparticles induced inflammatory and oxidative responses in the liver and kidney of rats. After ingestion and uptake, they are bio-distributed and accumulated in major organs such as the liver, spleen, and kidney^41^, posing systemic toxicity to animals. The liver and kidney function tests provides accurate information for diagnosis of diseases and ascertain the presence of infections^42^, where elevated activities of ALT and AST, and levels of creatinine and urea are indicative of hepatic and renal impairment respectively^43^.

In this present study, the integrity of the liver and kidney was compromised following administration of CeO_2_ and ZnO NPs. The damaging effect triggered by the administration of these nanoparticles was established via histopathological examination where alteration in cyto-architecture and infiltration of inflammatory cells in the liver and kidney of animals were observed. There are several mechanisms of hepatic and renal toxicity induced by nanoparticles, these include generation of ROS/free radicals, inflammatory processes and apoptosis^44^. In particular, metal-based nanoparticles have been found to induce oxidative stress due to generation of ROS in cells^44^. ROS which are oxygen ions or oxygen-containing radicals have been implicated in cytotoxicity and genotoxicity induced by toxicants, and nanoparticles have been known to play similar roles^45^. The significantly high levels of serum, hepatic and renal malondialdehyde (MDA) and very low levels of total sulphydryl (TSH) observed in NPs-treated rats confirmed induction of oxidative stress in the animals. These results are in tandem with the findings of ^46^ who reported alteration in homeostasis of total sulphydryl and lipid peroxidation in kidney of experimental rats, and ^44^ who showed that certain nanoparticles exert toxicity through the production of ROS such as superoxide anion, hydroxyl radicals and hydrogen peroxide. Superoxide dismutase (SOD) and catalase are first-line antioxidant enzymes against ROS. SOD is involved in the scavenging of superoxide anion radicals by converting them to hydrogen peroxide and oxygen, thus preventing peroxynitrite production and further damage^47^. In this study, the observed oxidative stress in the liver and kidney of nanoparticle-treated rats was accompanied by a significant decrease in hepatic and renal activities of SOD, catalase and glutathione peroxidise, and depletion of reduced glutathione. Decrease in antioxidant status of animals is a predisposing factor to the development of diseases and infections^48^. The enzyme: nitric oxide synthase (NOS) catalyzes the reaction that produces nitric oxide (NO) from l-arginine and oxygen^49^. As a vasodilator, nitric oxide is thought to play an important role in the homeostatic modulation of renal hemodynamics in both normotensive and hypertensive states^50^. Imbalance in redox homeostasis via elevated levels of ROS and reduced activities of antioxidant enzymes have been implicated in apoptosis through oxidative damage to intracellular proteins and DNA^51^. In addition, ROS scavenge nitric oxide, leading to the formation of peroxynitrite, which reduces NO bioavailability and induces oxidative damage in biomolecules^52^, all of which contributes to exacerbating inflammation in the tissues. In this study, inflammation of the liver and kidney was confirmed in rats treated with CeO_2_ and ZnO nanoparticles singly or combined, suggesting that the NPs can activate key processes, leading to inflammation. Various pro-inflammatory proteins also cause neutrophils to generate myeloperoxidase (MPO) and ROS during bacterial infection^53^. At the site of inflammation, activated neutrophils, monocytes, and tissue macrophages release MPO, which utilizes hydrogen peroxide to oxidize a variety of substrates, including pseudohalides to generate powerful oxidant like HOCl^54^. Elevated MPO, IL-1β and TNF-α observed in the nanoparticles-treated rats in this study further attest to induction of inflammation in tissues of the animals. Pharyngeal aspiration of ZnO-NPs induced infiltration of inflammatory cells in the lung of mice, but minimally induced *Nrf2*-dependent antioxidant enzymes^40^ and acute pulmonary exposure to CeO_2_ NPs causes oxidative stress, inflammation, and DNA damage in multiple major organs, including the lung, heart, liver, kidney, spleen, and brain^59^.

Caspase-9 is involved in the regulation of physiological cell death and degradation of worn out and damage cells. It is an important protein in the intrinsic pathway of apoptosis where it acts an initiator^55^. Serum activities of caspases-3 and -9 were not significantly altered in rats administered CeO_2_ and ZnO nanoparticles either singly or when combined. However, the increase in BAX in rats treated with CeO_2_ and ZnO nanoparticles singly and combined might suggest the induction of apoptosis. The Bcl-2 proto-oncogene family is known to regulate apoptosis^56^. Bcl-2 family members exist in two categories: the inhibitor of apoptosis (Bcl-2) and inducer of apoptosis (BAX)^56^. It is the relative abundance of pro-apoptotic and anti-apoptotic proteins that determines the susceptibility to cell death^57^. Our inability to determine the Bcl-2 levels in this study is one of the limitations and it is due to limited fund, as this research is self-sponsored.. In line with our results^58^, reported that CeO_2_ nanoparticles prevents the loss of mitochondria membrane potential, thereby preventing the apoptotic loss of cell viability. Due to their physico-chemical properties, nanoparticles have been found to invade tissues and organs (liver and kidney inclusive) through dermal exposure^40^. After nanoparticles have been administered, they can bio-accumulate in the liver, and followed by the kidney^40^. This necessitated to examine the effects of the two nanoparticles on the cytoarchitecture of the liver and kidney of the animals. In the liver of animals exposed to CeO_2_ nanoparticles, the sinusoids show mild infiltration of inflammatory cells and focal area of hepatocytes with necrosis (10–30%), while the moderate portal congestion and mild periportal infiltration of inflammatory cells observed in animals exposed to ZnO nanoparticles (Table 4) and the combination of CeO_2_ and ZnO is indicative of the ability of both nanoparticles. This observation was in tandem with Sabry et al. who reported that microscopic examination of histological sections of the liver showed pathological changes after injection of mice with cerium oxide in both low and high doses included dilatation in the central vein, congestion, infiltration of inflammatory, hyperplasia of hepatic cells, multinucleated cells and necrosis^60^. The control group showed intact hepatic architecture. The kidney parenchyma of animals exposed to CeO_2_ nanoparticles showed focal area of mild haemorrhage (6–10%) and fluid accumulation and the interstitial spaces show interstitial vascular congestion (Table 5) and moderate infiltration of inflammatory cells (scattered). In the ZnO nanoparticle treated rats, the interstitial spaces show moderate congestion (scattered), while in the combined nanoparticles group, the renal cortex showed glomeruli with dilated capsular spaces.

**Table 4:** The histological score of hepatic lesion.

Histological criteria	Severity	Description	Score
Necrosis	Absent	< 10%	0
	Mild	10–30%	1
	Severe	> 31%	2
Inflammation	None		0
	Mild	Scattered	1
	Severe	Diffuse	2
Congestion	None		0
	Mild	Scattered	1
	Severe	Condensed	2

**Table 5:** The histological score of renal lesion.

Histological criteria	Severity	Description	Score
Haemorrhage	Absent	0%	0
	Mild	< 10%	1
	Severe	11%	2
Inflammation	None		0
	Moderate	Scattered	1
	Severe	Diffuse	2
Congestion	None		0
	Moderate	Scattered	1
	Severe	Condensed	2

The present study confirmed earlier studies on the toxicity and adverse effect of exposure to CeO_2_ and ZnO nanoparticles, and also showed for the first time that exposure to the combined nanoparticles triggered hepato-renal toxicities via imbalance in oxidant /antioxidant levels. These effects caused oxidative stress and inflammation in the rats leading to alteration in the cyto-architecture of the liver and kidney.

## Data Availability

All datasets used or analyzed in the present research are readily accessible through the corresponding author at any time of acceptable request.

## References

[1] Mahmood B, Abbas R, Saman S (2021). Nanotechnology for inflammatory bowel disease management: Detection, imaging, and treatment. Sens. Biosens. Res..

[2] Mitchell MJ, Billingsley MM, Haley RM (2021). Engineering precision nanoparticles for drug delivery. Nat. Rev. Drug Discov..

[3] Harish V, Ansari M, Tewari D (2022). Nanoparticle and nanostructure synthesis and controlled growth methods. Nanomaterials.

[4] Palestino G, García-Silva I, González-Ortega O, Rosales-Mendoza S (2020). Can nanotechnology help in the fight against COVID-19*?*. Exp. Rev. Anti. Infect. Ther..

[5] Zhang C, Liu Z, Zhang Y (2020). “Iron free” zinc oxide nanoparticles with ion-leaking properties disrupt intracellular ROS and iron homeostasis to induce ferroptosis. Cell Death Dis..

[6] Tran MT, Nguyen HAT, Doan VD, Tran QH, Nguyen VC (2021). Biosynthesis of zinc oxide nanoparticles using aqueous piper betel leaf extract and its application in surgical sutures. J. Nanomater..

[7] Shcherbakov AB, Reukov VV, Yakimansky AV (2021). CeO_2_ Nanoparticle containing polymers for biomedical applications: A review. Polymers.

[8] Nabi-Afjadi M, Karami H, Goudarzi K (2021). The effect of vitamin D, magnesium and zinc supplements on interferon signaling pathways and their relationship to control SARS-CoV-2 infection. Clin. Mol. Allergy.

[9] Bhantana P, Timlin D, Rana MS (2020). How to cut down the gap between Zn requirement and supply of food chain and crop growth: A critical review. Int. J. Plant Anim. Environ. Sci..

[10] Siddiqui SA, Rashid MMO, Uddin MG (2020). Biological efficacy of zinc oxide nanoparticles against diabetes: A preliminary study conducted in mice. Biosci. Rep..

[11] Alhujaily M, Albukhaty S, Yusuf M (2022). Recent advances in plant-mediated zinc oxide nanoparticles with their significant biomedical properties. Bioengineering.

[12] Sander M, Sander M, Burbidge T (2020). The efficacy and safety of sunscreen use for the prevention of skin cancer. CMAJ.

[13] Mohd- Yusof H, Abdul Rahman N, Mohamad R, Hasanah ZU, Samsudin AA (2021). Antibacterial potential of biosynthesized zinc oxide nanoparticles against poultry-associated foodborne pathogens: An in vitro study. Animals.

[14] Anjum S, Hashim M, Malik S (2021). Recent advances in zinc oxide nanoparticles (ZnO NPs) for cancer diagnosis, target drug delivery, and treatment. Cancers.

[15] Gomaa E (2022). Microbial mediated synthesis of zinc oxide nanoparticles, characterization and multifaceted applications. J. Inorg. Organomet. Polym..

[16] Nyoka M, Choonara Y, Kumar P (2020). Synthesis of cerium oxide nanoparticles using various methods: Implications for biomedical applications. Nanomaterials.

[17] Ju X, Hubalek M, Šmíd B (2021). Poly(acrylic acid)-mediated synthesis of cerium oxide nanoparticles with variable oxidation states and their effect on regulating the intracellular ROS level. J. Mater. Chem. B.

[18] Alili L, Sack M, von Montfort C (2022). Downregulation of tumor growth and invasion by redox-active nanoparticles. Antioxid. Redox Signal.

[19] Casals G, PerramónCasals E, Portolés I (2021). Cerium oxide nanoparticles: A new therapeutic tool in liver diseases. Antioxidants (Basel, Switzerland).

[20] Wu Q, Xu S, Tan Y (2022). Exposure to ultrafine particles and childhood obesity: A cross-sectional analysis of the seven northeast cities (SNEC) study in China. Sci. Total Environ..

[21] Dominici F, Zanobetti A, Schwartz J (2022). Assessing adverse health effects of long-term exposure to low levels of ambient air pollution: Implementation of causal inference methods. Res. Rep. Health Eff. Inst..

[22] Campanale C, Massarelli C, Savino I (2020). A detailed review study on potential effects of microplastics and additives of concern on human health. Int. J. Environ. Res. Public Health.

[23] Felix LC, Ortega VA, Ede JD, Goss GG (2013). Physicochemical characteristics of polymer-coated metal-oxide nanoparticles and their toxicological effects on zebra fish (*Danio rerio*) development. Environ. Sci. Technol..

[24] Hirst SM, Karakoti A, Singh S (2013). Bio-distribution and in vivo antioxidant effects of cerium oxide nanoparticles in mice. Environ. Toxicol..

[25] Minarchick VC, Stapleton PA, Porter DW, Wolfarth MG, Çiftyürek E, Barger M, Nurkiewicz TR (2013). Pulmonary cerium dioxide nanoparticle exposure differentially impairs coronary and mesenteric arteriolar reactivity. Cardiovasc. Toxicol..

[26] Mohun AF, Cook LJ (1957). Simple method for measuring serum level of glutamate-oxaloacetate and glutamate-pyruvate transaminases in laboratories. J. Clin. Pathol..

[27] Reitman S, Frankel S (1957). A colorimetric method for the determination of serum level of glutamate-oxaloacetate and pyruvate transaminases. Am. J. Clin. Pathol..

[28] Jendrassik L, Grof P (1938). Simplified photometric methods for the determination of bilirubin. Biochem. J..

[29] Fawcett JK, Scott JE (1960). A rapid and precise method for the determination of urea. J. Clin. Pathol..

[30] Bartels H, Bohmer M, Heierli C (1972). Serum creatinine determination without protein precipitation. Clin. Chim. Acta.

[31] Lowry OH, Rosebrough NJ, Farr AL, Randall RJ (1951). Protein measurement with the Folin phenol reagent. J. Biol. Chem..

[32] Aebi, H. Catalase estimation. in *Methods of Enzymatic Analysis* (Bergmeyer, H.U. Ed.). Vol. 12. 673–680 (VerlagChemie/Academic Press Inc., 1974)

[33] McCord JM, Fridovich I (1969). Superoxide dismutase, an enzymatic function for erythrocuprein. J. Biol. Chem..

[34] Habig WH, Pabst MJ, Jakoby WB (1974). Glutathione-*S*-transferases. The first enzymatic step in mercapturic acid formation. J. Biol. Chem..

[35] Rotruck JT, Pope AL, Ganther HE, Swanson AB, Hafeman DG, Hoekstra WG (1973). Selenium: Biochemical role as a component of glutathione peroxidase. Science.

[36] Moron MS, Depierre JW, Mannervick B (1979). Levels of glutathione, glutathione reductase and glutathione-*s*-transferase activities in rat lung and liver. Biochem. Biophys. Acta..

[37] Buege J, Aust S (1978). Microsomal lipid peroxidation. Methods Enzymol..

[38] Palmer R, Ferrige A, Moncada S (1987). Nitric oxide release accounts for the biological activity of endothelium-derived relaxing factor. Nature.

[39] Trush M, Egner P, Kensler T (1994). Myeloperoxidase as a biomarker of skin irritation and inflammation. Food Chem. Toxicol..

[40] Janßen H, Angrisani N, Kalies S (2020). Bio-distribution, biocompatibility and targeted accumulation of magnetic nanoporous silica nanoparticles as drug carrier in orthopedics. J. Nanobiotechnol..

[41] Sani A, Cao C, Cui D (2021). Toxicity of gold nanoparticles (AuNPs): A review. Biochem. Biophys. Rep..

[42] Bertolini A, van de Peppel IP, Bodewes FAJA (2020). Abnormal liver function tests in patients with COVID-19: Relevance and potential pathogenesis. Hepatology.

[43] Shaban EE, Elbakry HFH, Ibrahim KS (2019). The effect of white kidney bean fertilized with nano-zinc on nutritional and biochemical aspects in rats. Biotechnol. Rep..

[44] Yu Z, Li Q, Wang J (2020). Reactive oxygen species-related nanoparticle toxicity in the biomedical field. Nanoscale Res. Let..

[45] Howard D, Sebastian S, Le QV (2020). Chemical mechanisms of nanoparticle radiosensitization and radioprotection: A review of structure–function relationships influencing reactive oxygen species. Int. J. Mol. Sci..

[46] Mahajan L, Verma PK, Raina R (2018). Alteration in thiols homeostasis, protein and lipid peroxidation in renal tissue following subacute oral exposure of imidacloprid and arsenic in Wistar rats. Toxicol. Rep..

[47] Ighodaro O, Akinloye OA (2018). First-line defense antioxidants-superoxide dismutase (SOD), catalase (CAT) and glutathione peroxidase (GPX): Their fundamental role in the entire antioxidant defense grid. Alex. J. Med..

[48] Forman HJ, Zhang H (2021). Targeting oxidative stress in disease: Promise and limitations of antioxidant therapy. Nat. Rev. Drug Discov..

[49] Förstermann U, Sessa WC (2012). Nitric oxide synthases: Regulation and function. Eur Heart J..

[50] Carlström M (2021). Nitric oxide signaling in kidney regulation and cardio-metabolic health. Nat. Rev. Nephrol..

[51] Tan BL, Norhaizan ME, Liew WP, Sulaiman RH (2018). Antioxidant and oxidative stress: A mutual interplay in age-related diseases. Front. Pharmacol..

[52] Corpas F, González-Gordo S, Palma J (2021). Nitric oxide (NO) Scaffolds the peroxisomal protein–protein interaction network in higher plants. Int. J. Mol. Sci..

[53] Goud P, Bai D, Abu-Soud H (2021). A multiple-hit hypothesis involving reactive oxygen species and myeloperoxidase explains clinical deterioration and fatality in COVID-19. Int. J. Biol. Sci..

[54] Alghsham RS, Satpathy SR, Bodduluri SR (2019). Zinc oxide nanowires exposure induces a distinct inflammatory response via CCL11-mediated eosinophil recruitment. Front. Immunol..

[55] Singh V, Khurana A, Navik U (2022). Apoptosis and pharmacological therapies for targeting thereof for cancer therapeutics. Science.

[56] Qian S, Wei Z, Yang W, Huang J, Yang Y, Wang J (2022). The role of BCL-2 family proteins in regulating apoptosis and cancer therapy. Front. Oncol..

[57] Chaudhry G, Akim A, Sung Y (2022). Cancer and apoptosis. Methods Mol. Biol..

[58] Akhtar M, Ahamed M, Alhadlaq H (2022). CeO_2_–Zn nanocomposite induced superoxide, autophagy and a non-apoptotic mode of cell death in human umbilical-vein-derived endothelial (HUVE) cells. Toxics.

[59] Nemmar A, Priya Y, Sumaya B, Mohamed F, Ali B (2017). Cerium oxide nanoparticles in lung acutely induce oxidative stress, inflammation, and DNA damage in various organs of mice. Oxid. Med. Cell. Longev..

[60] Sabry S, Hammid S, Jeber Al-Maliki AA, Al-Ali A (2023). Histopathological and enzymatic changes in male mice liver induced by CeO_2_ nanoparticle. Int. J. Pharm. Res..

